# Prevalence of severe fever with thrombocytopenia syndrome virus in animals in Henan Province, China

**DOI:** 10.1186/s40249-019-0569-x

**Published:** 2019-06-24

**Authors:** Xue-Yong Huang, Yan-Hua Du, Hai-Feng Wang, Ai-Guo You, Yi Li, Jia Su, Yi-Fei Nie, Hong-Xia Ma, Bian-Li Xu

**Affiliations:** 1 Henan Center for Disease Control and Prevention, Zhengzhou, China; 2 Henan Key Laboratory of Pathogenic Microorganisms, Zhengzhou, China

**Keywords:** Severe fever with thrombocytopenia syndrome virus, Epidemiological investigation, Animal, Reservoir host

## Abstract

Severe fever with thrombocytopenia syndrome (SFTS) is an emerging infectious disease caused by severe fever with thrombocytopenia syndrome virus (SFTSV). SFTSV has been found in humans, ticks and animals, and SFTS has high mortality and increasing prevalence in East Asia. In the study, the samples (heart, liver, lung, kidney, spleen, brain tissue and serum) were collected from 374 domestic animals and 241 wild animals in Pingqiao District and Xinxian County of Xinyang in Henan Province, China. 275 (44.72%, 275/615) animals were positive for anti-SFTSV antibodies, the anti-SFTSV antibodies positive ratios of domestic and wild animals were 43.58% (163/374) and 46.47% (112/241), respectively. There was no significant difference in domestic and wild animals, but significant differences were detected among different species of animals (*χ*^2^ = 112.59, *P* < 0.0001). Among 615 animals, 105 (17.07%, 105/615) animals were positive for SFTSV RNA, and only one SFTSV strain was isolated from heart tissue of a yellow weasel. The phylogenetic analysis shows that the sequence from animals belonged to the same group with viral sequences obtained from humans. The animals maybe play a reservoir host in maintaining the life cycle of SFTSV in nature.

**Electronic supplementary material:**

The online version of this article (10.1186/s40249-019-0569-x) contains supplementary material, which is available to authorized users.

## Multilingual abstracts

Please see Additional file 1 for translation of the abstract into the five official working languages of the United Nations.

## Background

Severe fever with thrombocytopenia syndrome (SFTS) is an emerging hemorrhagic fever and its pathogen is severe fever with thrombocytopenia syndrome virus (SFTSV). The disease has major clinical presentations that include fever, thrombocytopenia, leukocytopenia, gastrointestinal symptoms, neurological symptoms and a bleeding tendency, as well as less specific clinical manifestations [1]. The disease is widely expanding at global level. Between 2011 and 2014, 5352 suspected, probable and lab-confirmed cases of SFTS were reported in 23 provinces of China. Henan, Shandong, Hubei, Anhui, Liaoning, Zhejiang and Jiangsu reported 99.3% of those lab-confirmed cases [2–4]. SFTS cases had been reported in Japan and Republic of Korea. In Japan, 163 patients with SFTS were confirmed from the autumn of 2012 to October 2015. The number of SFTS cases reported in Republic of Korea had also increased in recent years, and were 36 (2013), 55 (2014) and 79 (2015) [5, 6].

SFTSV has a wide host range and infects in some animal species. The pooled seroprevalence of anti-SFTSV antibodies was 45.70% in goats and sheep, 36.70% in cattle, 29.50% in dogs, 9.60% in chickens, 3.20% in rodents, and 3.20% in pigs. SFTSV RNA was also detected in blood samples from several animal species, with a carriage rate varying from 0.23 to 26.31%, The highest carriage rate of SFTSV RNA was detected in cattle (up to 26.31%), followed by cats (17.46%), goats (9.10%), and rodents (8.44%), but there is no evidence that the virus can cause disease in animals [7, 8].

It is known that SFTSV can be detected in humans, various domestic animals and ticks. Although researchers had demonstrated in the laboratory that the SFTSV can be passed transstadially in the developmental stages of the ticks which can be as a host and reservoir of infection to spread SFTSV by biting humans and animals. But the prevalence of SFTSV infection among ticks collected from vegetation was quite low, suggesting that ticks alone may not be sufficient to maintain the virus in nature, so SFTSV must rely on other animals as storage hosts to circulate in nature [9]. This study was to explore the animal host reservoir range of SFTSV, and to elucidate SFTSV ecology in nature by detecting the anti-SFTSV antibodies and SFTSV RNA positive ratio of domestic animals and wild animals in the SFTS endemic region.

## Methods

### Research area and sample collection

The study was conducted in Pingqiao District and Xinxian County of Xinyang City in Henan Province, China, where had the most reported SFTS cases and was the most severe affected areas in China [10, 11]. From May 1, 2016 to April 30, 2018, the samples (serum, heart, liver, lung, kidney, spleen and brain tissue) were collected from 374 domestic animals (including 48 cattle, 76 sheep, 44 dogs, 63 pigs, 82 chickens and 61 ducks) and 241 wild animals (including 45 yellow weasels, 73 hares, 52 wild mice, 15 hedgehogs, five squirrels, three wild boars, four badgers, 23 rock pigeons, 14 pheasants and seven turtledoves) (Table 1). All samples were transported under refrigeration to Henan Center for Disease Control and Prevention (Henan CDC) and kept in a freezer at − 80 °C.

**Table 1:** The results of SFTSV RNA and antibodies in specimens of animals by real-time RT-PCR or ELISA assay

Animal species	Number of Animal (Positive rate %)	Heart (Positive rate %)	Liver (Positive rate %)	Spleen (Positive rate %)	Lungs (Positive rate %)	Kidney (Positive rate %)	Serum (Positive rate %)	
Domestic animals							SFTSV RNA	SFTSV antibodies
Cattles	48 (18.75%)	1 (2.08%)	2 (4.17%)	5 (10.42%)	3 (6.25%)	3 (6.25%)	1 (2.08%)	47 (97.92%)
Sheep	76 (15.79%)	3 (3.95%)	4 (5.26%)	7 (9.21%)	2 (2.63%)	4 (5.26%)	0	53 (69.74%)
Dogs	44 (13.64%)	0	2 (4.55%)	3 (6.82%)	1 (2.27%)	1 (2.27%)	0	30 (68.18%)
Pigs	63 (11.11%)	3 (4.76%)	1 (1.59%)	4 (6.35%)	1 (1.59%)	0	0	2 (3.17%)
Chickens	82 (19.51%)	9 (10.98%)	7 (8.54%)	4 (4.82%)	10 (12.20%)	0	0	19 (23.17%)
Ducks	61 (14.75%)	3 (4.92%)	5 (8.20%)	2 (3.28%)	5 (8.20%)	1 (1.64%)	0	12 (19.67%)
Total	374 (15.76%)	19 (5.08%)	21 (5.61%)	25 (6.68%)	22 (5.88%)	9 (2.41%)	1 (0.23%)	163 (43.58%)
Wild animals								
Yellow weasels	45 (37.78%)	2 (4.44%)	4 (8.89%)	11 (24.44%)	3 (6.67%)	6 (13.33%)	1 (2.22%)	41 (91.11%)
Hares	73 (21.92%)	3 (4.11%)	1 (1.37%)	13 (17.81%)	3 (4.11%)	2 (2.74%)	2 (2.74%)	46 (63.01%)
Wild mice	52 (3.85%)	0	2 (3.85%)	0	0	0	0	4 (7.69%)
Hedgehogs	15 (13.33%)	0	0	2 (13.33%)	0	0	0	6 (40.00%)
Squirrels	5	0	0	0	0	0	0	0
Wild boars	3	0	0	0	0	0	0	0
Badgers	4	0	0	0	0	0	0	0
Rock pigeons	23 (21.74%)	3 (13.04%)	2 (8.70%)	0	1 (4.35%)	0	0	8 (34.78%)
Pheasants	14 (28.57%)	0	2 (14.29%)	0	3 (21.43%)	0	0	6 (42.86%)
Turtledoves	7	0	0	0	0	0	0	1 (14.29%)
Total	241 (19.09%)	8 (3.32%)	11 (4.56%)	26 (10.79%)	10 (4.15%)	8 (3.32%)	3 (1.24%)	112 (46.47%)

Animal positive rate: at least one sample from one animal was positive for SFTSV RNA by real-time RT-PCR, the samples include heart, liver, spleen, lungs and kidney tissue

Detection methods: the heart, liver, spleen, lungs and kidney tissues were tested by real-time RT-PCR; the serum samples were tested by real-time RT-PCR and ELISA assay

*Abbreviations*: *SFTSV* Severe fever with thrombocytopenia syndrome virus, *RT-PCR* Reverse transcription polymerase chain reaction, *ELISA* Enzyme-linked immunosorbent assay, *RNA* Ribonucleic acid

### Enzyme-linked immunosorbent assays (ELISA)

Total SFTSV antibodies (including IgG and IgM) were detected in serum samples using a double-antigen sandwich ELISA kit (Xinlianxin Biomedical Technology CO., LTD, Wuxi, Jiangsu, China) according to the manufacturer’s protocol. For this ELISA kit, 100 μl of serum samples were diluted to 1∶5, and positive and negative controls were added. Samples were incubated at 37 °C for 30 min, washed with phosphate-buffered saline (PBS), and 100 μl of horseradish peroxidase-conjugated nucleoprotein was added. Plates were developed by using a substrate solution after samples were incubated and washed. The chromogenic reaction was stopped by sulfuric acid, and absorbance was identified at 450 nm. The test results were determined to be valid if the criteria for the positive and the negative controls were fulfilled [12, 13].

### Real-time reverse transcription polymerase chain reaction (RT-PCR) detection

The animal tissue samples (0.5–1 cm^3^) were ground by Tissuelyser (Mixer Mill MM 400, Retsch GmbH, Haan, Germany). Total RNA was extracted from serum samples and tissue-grinding solution using a QIAamp viral RNA mini Kit (Qiagen, Hilden, Germany), according to the manufacturer’s instructions. RNA was eluted in a final volume of 60 μl elution buffer and used immediately or stored at − 80 °C. RNA from each sample was examined by real-time RT-PCR Kit (Beijing BGI-GBI Biotech Co., Ltd. Beijing, China). The conditions for real-time RT-PCR reaction were as follows: 50 °C for 30 min, 95 °C for 15 min, 40 cycles of 95 °C for 15 s, and 60 °C for 45 s. Criteria for a positive result was cycle number (Ct) < 38, according to the instructions from the real-time RT-PCR Kit.

### Virus isolation and sequencing

All positive samples (serum, tissue-grinding solution) for SFTSV RNA were used to inoculate Vero E6 cells. After processing, 100 μl of samples was inoculated into a 25 cm^2^ flask containing Vero E6 cell monolayers. Tubes were incubated at 37 °C for 14 days and changed media after 7 days. All cultures were monitored daily for the cytopathic effect (CPE). Each sample underwent at least three cell culture passages in Vero E6 cells before being considered negative [14]. Both virus-infected cells and negative control cells were examined for SFTSV by real-time RT-PCR at each passage. The whole genome sequences of SFTSV were amplified using primers described in previous studies by RT-PCR [15]. The RT-PCR products were sent to Sangon Biotech Co., Ltd. (Shanghai, China) for DNA sequencing with an automated ABI 3730 DNA sequencer (Applied Biosystems, Foster City, CA, USA).

### Genome alignments and phylogenetic analysis

The genomes of SFTSV isolates were compiled using the SeqMan program in the Lasergene software package (DNASTAR, Version 2.0, Madison, WI, USA). The percentage similarities of nucleotide identity were calculated using the Clustalx software (Version 2.0, European Bioinformatics Institute [EMBLEBI], Cambridge, UK). Molecular phylogenetic analysis was conducted by using the maximum likelihood (ML) method based on the Kimura 2-parameter model in the molecular evolutionary genetics analysis (MEGA) 5 software (available at: http://mega.software.informer.com/5.0/) [16]. The tree with the highest log likelihood was shown, and the percentage of trees in which the associated taxa clustered together was shown next to the branches [11]. The available nucleotide sequences of genome segments of SFTSV isolates from GenBank were analysed, together with the newly generated genes of SFTSV in this study, and the phylogenetic trees were constructed to understand the evolutionary characterization of SFTSV.

### Statistical analysis

All statistical analyses were performed using SAS (Version 9.13, SAS Institute Inc., Cary, NC, US). Count data was analyzed by *χ*^2^ test or Fisher’s exact test. A significant difference was considered with a *P* value less than 0.05.

### Ethical clearance

The research was approved by the Ethical Committee of Henan CDC (Reference number: 2016-KY-002-02). The collection of wild animals was permitted by Pingqiao District and Xinxian County Forestry Bureau. Animals were captured by hunters, who were supplied with safety protection.

## Results

### Anti-SFTSV antibody among animals

All serum samples were tested for anti-SFTSV total antibodies using an ELISA assay. Two hundred seventy-five animals (44.72%, 275/615) were positive for anti-SFTSV antibodies; the positive ratios of domestic and wild animals were 43.58% (163/374) and 46.47% (112/241), respectively, there was no significant difference between domestic and wild animals (*χ*^2^ = 0.1884, *P* > 0.05), The positive ratio of the mammals (53.50%, 229/428) was higher than that of birds (24.60%, 46/187) (*χ*^2^ = 18.40, *P* < 0.0001). The higher positivity rate of the antibodies in domestic animals ware cattle (97.92%, 47/48), sheep (69.74%, 53/76) and dogs (68.18%, 30/44), the lowest positive rate in domestic animals is pigs (3.17%, 2/63). In the wild animals, yellow weasels (91.11%, 41/45) and hares (63.01%, 46/73) were higher than other animals, there are three animals (squirrels and wild boars and badgers) the positive rates were zero. There were significant differences in different species of animal (*χ*^2^ = 112.59, *P* < 0.0001) (Table 1).

### Real-time PCR for SFTSV on animal samples

In 615 animal serum samples, four samples were classified as positive based on real-time RT-PCR Kit criteria (one cattle, one yellow weasel and two hares). Additionally, 105 out of 615 (17.07%) animals were positive for SFTSV RNA, in which at least one organ was positive according to real-time RT-PCR. The brains were negative for SFTSV RNA in all animals. The positive rate of domestic animals and wild animals were 15.76% (59/374) and 19.09% (46/241), respectively. There were no significant differences between domestic animals and wild animals (*χ*^2^ = 0.7994, *P* > 0.05). There were no significant differences in the positive rates of SFTSV RNA among animal species (*χ*^2^ = 23.77, *P* > 0.05), but the positive rate was higher in the domestic cattle (18.75%, 9/48), chickens (19.51%, 16/82) and sheep (15.79%, 12/76), and in the wild animals yellow weasels (37.78%, 17/45), pheasants (28.57%, 4/14), hares (21.92%, 16/73) and rock pigeons (21.74%, 5/23). There were significant differences in the positive rates among organs of animals (*χ*^2^ = 105.70, *P* < 0.0001), the positive rate was the highest in spleen (8.29%, 51/615), and the lowest in kidney (2.76%, 17/615) (Table 1).

### Sequence analysis of isolates

One strain SFTSV was isolated and confirmed from heart tissue of a yellow weasel in this study by real-time RT-PCR. The genomes of the SFTSV isolates were sequenced and submitted to GenBank (Accession No. MF574211, MF574212, MF574213). The nucleotide sequences of this SFTSV were aligned using the basic local alignment search tool (BLAST) at the National Centre for Biotechnology Information (NCBI) (https://blast.ncbi.nlm.nih.gov/Blast.cgi). BLAST results showed that this isolate strain was related closely to known sequences, with 96.64 to 99.91% nucleotide identity for the complete L segments, 96.15 to 99.67% nucleotide identity for the complete M segments, and 98.51 to 99.89% nucleotide identity for the complete S segments.

**Fig. 1:**
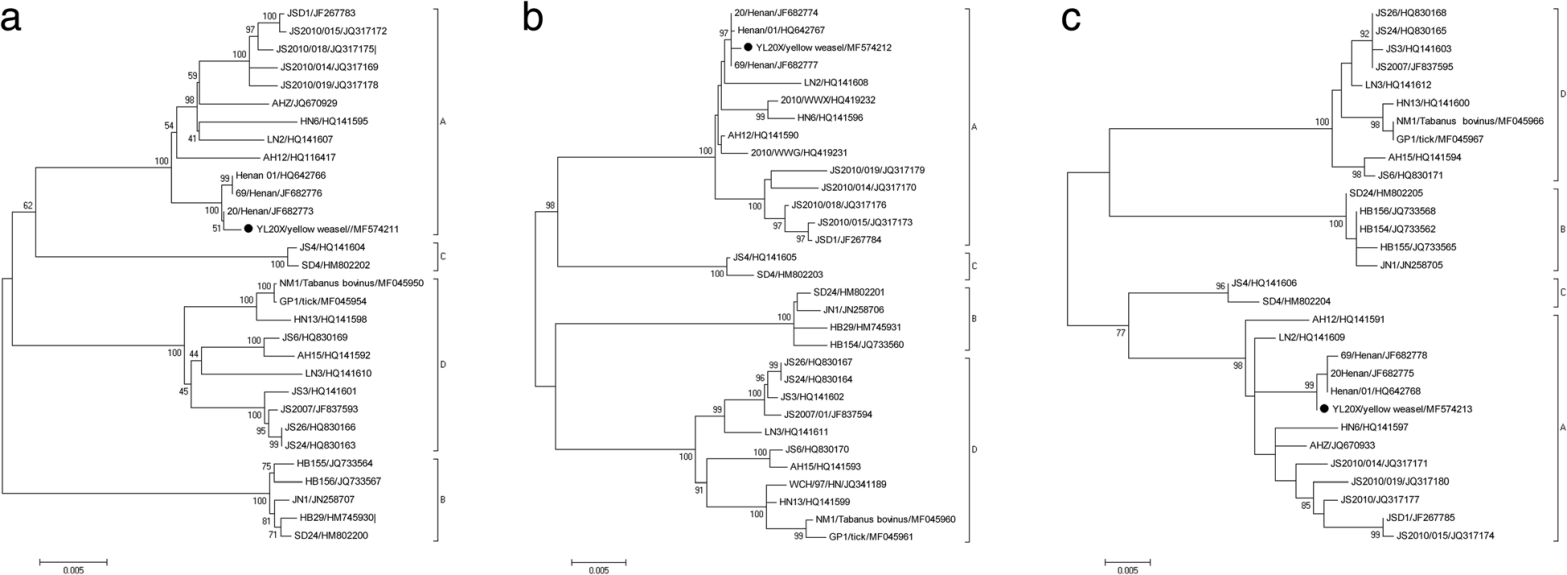
Phylogenetic analyses of SFTSV based on L, M, S segment sequences. The tree shows the comparison between the SFTSV strains of study and reference strains. The circle symbol represents SFTSV strains isolated from yellow weasel in this study

Phylogenetic trees were constructed with the sequences of 28 SFTSV strains from GenBank. Based on the alignment of L, M, S segments sequences for phylogenetic analysis, these SFTSV strains were classified into four groups (A, B, C and D). The SFTSV strain from animals belonged to Group A with viral strains obtained from humans in Henan Province (Fig.1).

## Discussion

Serological epidemiology of SFTSV was surveyed among domesticated animals in many previous studies. SFTSV antibody positive rate had a larger difference in the same animal species among different epidemic areas, but sheep, cattle, dogs and chickens had very high positive rate and high levels of SFTSV antibodies [17–20]. The SFTSV antibody positive rate was over 90% in cattle and 60% in sheep and dogs in this study. Liu, et al. and Li, et al. had investigated seropositive rate of SFTSV in rodents and hedgehogs by ELISA, and showed SFTSV antibody positive rate was low in these wild animals [21, 22]. In this study, the SFTSV antibody positive rate was similar with previous studies in domesticated animals, but was high in wild animals, especially in yellow weasels and hares. The SFTSV antibody positive rate was more than 90% in yellow weasels and 60% in hares. The incidence of SFTSV infection was correlated to the life style of the animals. This may be due to the free-range life style of domesticated cattle, goats, dogs and wild yellow weasels, hares, so these animals expose to a greater risk of tick bites than other animals, for example, pigs are usually captivity.

The results of this study showed that SFTSV-carrying rate was 17.07% among domestic animals and wild animals in epidemic areas in Henan, China, by real-time RT-PCR, and one SFTSV strain was isolated from a yellow weasel. The PCR positive rate of all animals was higher than all previous studies because the collection of sample types was different from them. In this study, the serum, heart, liver, lung, kidney, spleen and brain tissue samples were collected from animals. One organ from an animal was positive by real-time RT-PCR, the animal was judged as carrying SFTSV, but SFTSV RNA was only detected in serum samples from animals in previous studies. A total of 6637 blood samples from 11 animal species were testing for SFTSV RNA in twelve published articles, while the carriage rates of SFTSV RNA ranged from 0.23 to 26.31% [7] (Additional file 2: Table S1), 2.08% in cattle, 2.22% in yellow weasel, (2.74% on hare (in this survey, the results were similar with those previous studies. The positive rate of domestic animals and wild animals were no significant differences, and there were no significant differences between animal species. SFTSV RNA was detected in heart, liver, spleen, lungs, kidney from cattle, goats, wild yellow weasels and hares, which were more susceptible to infection and carry SFTSV. The results showed that some animals can carry SFTSV but no any specific clinical signs of illness.

The previous animal experiments showed that newborn mice, mitomycin-treated mice, and type I IFN-deficient mice are available as models for the fatal illness caused by SFTSV infection [23–25]. Immunocompetent adult mice, hamsters, and macaques were not susceptible to SFTSV and were asymptomatic, the level of white blood cell were slightly low among experimental animals and platelet counts at one time point; SFTSV load substantially decreased in blood on day 3 or 4 after inoculation; SFTSV RNA was detected in the spleen, liver, kidney; SFTSV-specific IgM and IgG antibodies were detected in sera [26–29].

Natural infection study showed goats were infected by ticks in the SFTS-endemic region. The goats were viremia over a very short period (less than 24 h) after viral infection, soon occupied by a timely mounting antibody response which effectively controlled the infection. The whole cohort did not show any specific clinical signs of illness, and all survived infection [9, 30]. The animals were infected in the laboratory or SFTS-endemic region, and viremia persisted for a short period, but SFTSV antibody can last for a long time, so detection results of antibody and RNA were greatly different in blood of animals with SFTSV infection. The domestic animals and wild animals may be intermediate animal hosts during SFTSV amplification in endemic area.

This study has two limitations. First, animal selection lacked uniformity in number, especially some wild animals were lesser and captured difficultly in nature. Second, animal species can’t be randomly selected to capture.

## Conclusions

The most animals were infected by SFTSV and some carry SFTSV in epidemic areas in China. 44.72% animals were positive for anti-SFTSV antibodies and 17.07% animals were positive for SFTSV RNA in Xinyang in Henan Province. The animals maybe play a reservoir host in maintaining the life cycle of SFTSV in nature.

## Additional files

Additional file 1: Multilingual abstracts in the five official working languages of the United Nations. (PDF 402 kb)

Additional file 2: **Table S1.** SFTSV RNA positivity as detected in the different animal species from published articles. (DOC 37 kb)

## Data Availability

Not applicable.

## Abbreviations

CDC: Center for Disease Control and Prevention
CPE: Cytopathic effect
ELISA: Enzyme-linked immunosorbent assay
ML: Maximum likelihood
PBS: Phosphate-buffered saline
RNA: Ribonucleic acid
RT-PCR: Reverse transcription polymerase chain reaction
SFTS: Severe fever with thrombocytopenia syndrome
SFTSV: Severe fever with thrombocytopenia syndrome virus
