# Supplementary material for: Prevalence of severe fever with thrombocytopenia syndrome virus in animals in Henan Province, China
Source: Infect Dis Poverty. 2019 Jun 24;8:56. doi: 10.1186/s40249-019-0569-x (PMC6589873; doi:10.1186/s40249-019-0569-x)
Supplement: Supplementary file 1 — Multilingual abstracts in the five official working languages of the United Nations. (PDF 402 kb) [file 40249_2019_569_MOESM1_ESM.pdf]

Translation of the abstract into the five official working languages of the United Nations

انتشار فيروس متلازمة ارتفاع الحرارة الشديد المترافق مع نقص الصفيحات عند الحيوانات في مقاطعة هيناي في الصين

Xue-Yong Huang, Yan-Hua Du, Hai-Feng Wang, Ai-Guo You, Yi Li, Jia Su, Yi-Fei Nie, Hong-Xia Ma and Bian-Li Xu

الملخص

تعتبر متلازمة ارتفاع الحرارة الشديد المترافق مع نقص الصفيحات (SFTS) مرضاً خمجياً حديث الظهور يسببه فيروس متلازمة ارتفاع الحرارة الشديد المترافق مع نقص الصفيحات (SFTSV). تم العثور على فيروس (SFTS) عند الإنسان، القراد والحيوانات، ولهذه المتلازمة معدل وفيات مرتفع وانتشار متزايد في شرق آسيا. في الدراسة، تم اعتيان عينات نسيجية (من القلب، الكبد، الرئة، الكلية، الطحال، الدماغ ومصل الدم) من ٣٧٤ حيوان داجن و ٢٤١ حيوان بري في منطقة بينجكاو ومقاطعة شينشيان التابعتين لمدينة شينيانغ في محافظة خينان في الصين. كان 275 (%44.72, 615/275) حيواناً إيجابيين لأضداد (anti-SFTSV)، وبلغت نسبة إيجابية أضداد (anti-SFTSV) للحيوانات الداجنة والبرية %43.58 (374/163) و%46.47 (241/112), تباعاً. لم يكن هناك اختلاف مهم بين الحيوانات الداجنة والبرية، ولكن وجدت اختلافات مهمة بين الأجناس المختلفة للحيوان الواحد (χ^2 = 112.59, P < 0.0001). من بين 615 حيوانًا، كان 105 حيوانات (%17.07, 615/105) إيجابيي الـ (SFTSV RNA) وتمّ عزل سلالة واحدة فقط من فيروس الـ(SFTS) من النسيج القلبي لابن عرس أصفر. يظهر التحليل التطوري أنّ التسلسل الذي حُصل عليه من الحيوانات ينتمي إلى نفس المجموعة التي تنتمي إليها التسلسلات الفيروسية المأخوذة من البشر. ربما تلعب الحيوانات دور مضيف احتياطي للحفاظ على دورة حياة فيروس الـ(SFTS) في الطبيعة.

Translated from English version into Arabic by asel Qarabash, Revised by Ahmed Ibrahim, through

TRANSLATORS WITHOUT BORDERS

动物血小板减少综合征患病率与传播研究

Xue-Yong Huang, Yan-Hua Du, Hai-Feng Wang, Ai-Guo You, Yi Li, Jia Su, Yi-Fei Nie, Hong-Xia Ma and Bian-Li Xu

摘要

发热伴血小板减少综合征病毒引起的发热伴血小板减少综合征是一种新发传染病。目前在人、蜱和动物标本中已检测到发热伴血小板减少综合征病毒，该新发传染病具有较高的死亡率，且流行区域逐渐扩大。本研究在中国发热伴血小板减少综合征疫情高流行地区河南省信阳市平桥区和新县采集了 374 只家养动物和 241 只野生动物的心、肝、肺、肾、脾、脑组织和血清标本。实验室检测 275 (44.72%, 275/615) 只动物血清标本发热伴血小板减少综合征病毒抗体阳性，其中家养动物阳性率 43.58% (163/374)，野生动物抗体阳性率 46.47% (112/241)，家养动物和野生动物之间阳性率无显著差异，但不同种类动物之间存在显著差异

(χ^2 = 112.59, P < 0.0001)。在总共 615 只动物中，检测发热伴血小板减少综合征病毒核酸阳性 105 (17.07%, 105/615)只，并从 1 只野生动物黄鼠狼心脏标本中分离到 1 株发热伴血小板减少综合征病毒。系统进化分析显示发热伴血小板减少综合征病毒可在动物、蜱和人之间进行循环传播，证实动物可作为发热伴血小板减少综合征病毒的自然储存宿主。

Translated from English version into Chinese by Xue-Yong Huang,

Prévalence du virus responsable du syndrome de fièvre sévère avec thrombocytopénie chez virus in Animals in Henan Province, Chine

Xue-Yong Huang, Yan-Hua Du, Hai-Feng Wang, Ai-Guo You, Yi Li, Jia Su, Yi-Fei Nie, Hong-Xia Ma et Bian-Li Xu

Résumé

Le syndrome de fièvre sévère avec thrombocytopénie (SFTS) est une maladie infectieuse émergente causée par le virus du syndrome de fièvre sévère avec thrombocytopénie (SFTSV). Le SFTSV a été identifié chez les humains, les tiques et les animaux, et le SFTS présente un taux élevé de mortalité et une prévalence croissante en Asie de l'Est. Dans cette étude, les échantillons (cœur, foie, poumon, rein, rate, tissu cérébral et sérum) ont été collectés chez 374 animaux domestiques et 241 animaux sauvages dans le District de Pingqiao et le Comté Xinxian de Xinyang dans la Province de Henan, Chine. 275 (44,72%, 275/615) animaux étaient positifs aux anticorps anti-SFTSV, les rapports positifs des anticorps anti-SFTSV des animaux domestiques et sauvages étaient respectivement de 43,58% (163/374) et 46,47% (112/241). Il n'y avait pas de différence significative entre les animaux domestiques et sauvages, mais des différences significatives ont été détectées entre différentes espèces d'animaux ($\chi2$ = 112,59 ; P < 0,0001).Parmis 615 animaux, 105 (17,07%, 105/615) animaux étaient positifs à l'ARN SFTSV, et seulement une souche de SFTSV a été isolée à partir d'un tissu cardiaque de vison sibérien.L'analyse phylogénétique montre que la séquence provenant d'animaux appartient au même groupe que celui des séquences virales provenant des humains. Il se peut que les animaux jouent un rôle d'hôte réservoir dans le maintient du cycle de vie du SFTSV dans la nature.

Translated from English version into French by Eva De Nadaï, Revised by Ahmad Dabaghzadeh, through

TRANSLATORS WITHOUT BORDERS

Распространенность вируса тяжелой лихорадки с тромбоцитопеническим синдромом среди животных в провинции Хэнань, Китай

Сюэ-Юн Хуан, Янь-Хуа Ду, Хай-Фэн Ван, Ай-го Ю, И Ли, Цзя Су, И-Фэй Не, Хун-Ся Ма и Бянь-Ли Сюй

Аннотация
Тяжелая лихорадка с тромбоцитопеническим синдромом (SFTS) — это развивающееся инфекционное заболевание, вызванное вирусом тяжелой лихорадки с тромбоцитопеническим синдромом (SFTSV). SFTSV был обнаружен у людей, клещей и животных, а SFTS отличается высокой смертностью и увеличением распространенности в Восточной Азии. В ходе исследования были собраны образцы (сердце, печень, легкие, почки, селезенка, мозговая ткань и сыворотка) у 374 домашних животных и 241 дикого животного в районе Пинцяо и уезде Синьянь провинции Хэньань в Китае. У 275 (44,72 %, 275/615) из всех животных были обнаружены антитела к SFTSV, соотношение положительных антител к SFTSV среди домашних и диких животных составило 43,58 % (163/374) и 46,47 % (112/241), соответственно. Существенных различий между домашними и дикими животными не было, но были обнаружены значительные различия между различными видами животных (χ^2 = 112,59, P < 0.,0001). Среди 615 животных у 105 (17,07 %, 105/615) животных была обнаружена РНК SFTSV, и только один штамм SFTSV был выделен из сердечной ткани желтой ласки.Филогенетический анализ показывает, что последовательность, выделенная у животных, принадлежала к той же группе вирусных последовательностей, полученных от человека. Животные могут играть роль резервуара в поддержании жизненного цикла SFTSV в природе.

Translated from English version into Russian by Michael Orlov, Revised by Anna Kukharchuk, through

TRANSLATORS
WITHOUT BORDERS

La prevalencia del virus del síndrome de la fiebre severa con trombocitopenia en animales en la provincia Henan, China

Xue-Yong Huang, Yan-Hua Du, Hai-Feng Wang, Ai-Guo You, Yi Li, Jia Su, Yi-Fei Nie, Hong-Xia Ma y Bian-Li Xu

Resumen
El síndrome de la fiebre severa con trombocitopenia (SFTS, por sus siglas en inglés) es una enfermedad infecciosa emergente causada por el virus de la fiebre severa con trombocitopenia (SFTSV, por sus siglas en inglés). El SFTSV se ha encontrado en seres humanos, garrapatas y animales, y el SFTS tiene una elevada tasa de mortalidad y una prevalencia creciente en el este de Asia. En el estudio, se recogieron muestras (corazón, hígado, pulmones, riñones, bazo, tejido cerebral y suero) de 374 animales domésticos y 241 animales salvajes en el distrito de Pingqiao y

en el condado Xinxian de Xinyang en la provincia de Henan, China. 275 (44,72 %, 275/615) animales resultaron positivos para anticuerpos contra el SFTSV; el índice de anticuerpos positivos contra el SFTSV en animales domésticos y en animales salvajes fue del 43,58 % (163/374) y del 46,47 % (112/241), respectivamente. No se encontraron diferencias considerables entre animales domésticos y animales salvajes, pero sí se detectaron entre diferentes especies de animale s (χ^2 = 112,59, $P < 0{,}0001$). De 615 animales, 105 (17,07 %, 105/615) resultaron positivos para el SFTSV ARN y solo se aisló una cepa del SFTSV en el tejido cardíaco de una comadreja de vientre amarillo. El análisis filogenético muestra que la secuencia de los animales pertenecía al mismo grupo que las secuencias virales obtenidas de los seres humanos. Es posible que los animales actúen como huésped reservorio para mantener el ciclo biológico del SFTSV en la naturaleza.

Translated from English version into Spanish by Laura García Sánchez, Revised by Maria Paula Gorgone, through

TRANSLATORS
WITHOUT BORDERS
