# Supplementary material for: Prevalence of severe fever with thrombocytopenia syndrome virus in animals in Henan Province, China
Source: Infect Dis Poverty. 2019 Jun 24;8:56. doi: 10.1186/s40249-019-0569-x (PMC6589873; doi:10.1186/s40249-019-0569-x)
Supplement: Supplementary file 2 — Table S1. SFTSV RNA positivity as detected in the different animal species from published articles. (DOC 37 kb) [file 40249_2019_569_MOESM2_ESM.doc]

Additional file 2: Table S1. SFTSV RNA positivity as detected in the different animal species from published articles

| NO. | First author | Article title | Country | Journal name | Publication year | Species |
| --- | --- | --- | --- | --- | --- | --- |
| 1 | Kang JG, et al. | Prevalence of severe fever with thrombocytopenia syndrome virus in black goats (Capra hircus coreanae) in the Republic of Korea. | Korea | Ticks Tick Borne Dis | 2018 | Goats and sheep |
| 2 | Yu KM, et al. | Seroprevalence and genetic characterization of severe fever with thrombocytopenia syndrome virus in domestic goats in South Korea. | Korea | Ticks Tick Borne Dis | 2018 | Goats and sheep |
| 3 | Kang JG, et al. | Molecular detection of severe fever with thrombocytopenia syndrome virus in Korean domesticated pigs. | Korea | Vector Borne Zoonotic Dis | 2018 | Pigs |
| 4 | Lee, SH, et al. | Molecular detection and phylogenetic analysis of severe fever with thrombocytopenia syndrome virus in shelter dogs and cats in the Republic of Korea. | Korea | Ticks Tick Borne Dis | 2017 | Dogs and Cats |
| 5 | Hwang J, et al. | Molecular detection of severe fever with thrombocytopenia syndrome virus (SFTSV) in feral cats from Seoul, Korea. | Korea | Ticks Tick Borne Dis | 2017 | Cats |
| 6 | Oh SS, et al. | Detection of Severe Fever with Thrombocytopenia Syndrome Virus from Wild Animals and Ixodidae Ticks in the Republic of Korea. | Korea | Vector Borne Zoonotic Dis | 2016 | Deer and Boar |
| 7 | Xiong JF, et al. | Survey on ticks and host animals of severe fever with thrombocytopenia syndrome virus in Huanggang, Hubei province. | China | Chinese Journal of Vector Biology and Control | 2016 | Cattle, Goats and sheep |
| 8 | Li Z, et al. | Ecology of the Tick-Borne Phlebovirus Causing Severe Fever with Thrombocytopenia Syndrome in an Endemic Area of China. | China | PLoS Negl Trop Dis | 2016 | Rodents, Shrews, and Hedgehogs |
| 9 | Gong LF, et al. | Prevalence and homology analysis on human and animals severe fever with thrombocytopenia syndrome virus infection in Yantai of Shandong province. | China | Chinese Journal of Epidemiology | 2014 | Cattle, Dogs, Goats and sheep |
| 10 | Liu JW, et al. | Prevalence of SFTSV among Asian House Shrews and Rodents, China, January–August 2013. | China | Emerg Infect Dis | 2014 | rodents and shrews |
| 11 | Niu G, et al. | Severe fever with thrombocytopenia syndrome virus among domesticated animals, China. | China | Emerg Infect Dis | 2013 | Cattle, Dogs, Pigs, Chickens, Goats and sheep |
| 12 | Ge HM, et al. | Survey of SFTSV carrying situation in rodents in epidemic area of Donghai county. | China | Jiangsu J Prev Med | 2012 | Rodents and Shrews |
